# An Ultrasensitive Chemiluminescence Biosensor for Carcinoembryonic Antigen Based on Autocatalytic Enlargement of Immunogold Nanoprobes

**DOI:** 10.3390/s121217320

**Published:** 2012-12-13

**Authors:** Minjia Hao, Zhanfang Ma

**Affiliations:** Department of Chemistry, Capital Normal University, Beijing 100048, China

**Keywords:** carcinoembryonic antigen, flow injection chemiluminescence, gold nanoparticle, enlargement

## Abstract

A sensitive flow injection chemiluminescence assay for carcinoembryonic antigen (CEA) detection based on signal amplification with gold nanoparticles (NPs) is reported in the present work. The sandwich system of CEA/anti-CEA/goat-anti-mouse IgG functionalized Au nanoparticles was used as the sensing platform. In order to improve detection sensitivity, a further gold enlargement step was developed based on the autocatalytic Au deposition of gold nanoprobes via the reduction of AuCl_4_^−^ to Au^0^ on their surface in the presence of NH_2_OH·HCl. AuCl_4_^−^, which is a soluble product of gold nanoprobes, served as an analyte in the CL reaction for the indirect measurement of CEA. Under optimized conditions, the CL intensity of the system was linearly related to the logarithm of CEA concentration in the range of 100 pg·mL^−1^ to 1,000 ng·mL^−1^, with a detection limit of 20 pg·mL^−1^.

## 1. Introduction

The development of highly sensitive methods for detecting cancer biomarkers has great significance for predicting cancer in early stage in modern biochemical and biomedical research [1–3]. Generally, high sensitivity can often be obtained using a signal amplification procedure. Many signal amplification strategies have been reported for sensitive bioanalyte quantification, such as rolling circle amplification [4], avidin-biotin amplification [5], and exponential isothermal amplification [6]. However, these approaches usually require extremely complex reagents and intensive protocols. Recently, amplification of immunoassay signals by exploiting the properties of gold nanoparticles (AuNPs) for catalytic growth has emerged as a field attracting significant attention. The commonly used example is silver enhancement, in which AuNPs promote the reduction of silver ions and their deposition on the surface of AuNPs, thereby resulting in further growth of the AuNPs [7]. But it is sensitive to pH, natural light, and chloride ions [8]. Instead of silver staining, using gold salts for catalytic enlargement of AuNPs has solved these issues [9]. This alternative approach has been successfully exploited to develop the detection of IgG by the naked eye [10], target DNA by electrochemical means [11,12] and others [13–15].

Chemiluminscence immunoassay (CLIA), which was first reported in the late 1970s [16], simultaneously combines the high sensitivity of chemiluminescence analysis and the good specificity of immunoassays. Since most biomolecules (such as tumor markers) have no native CL emission, CLIA quantitation is generally realized by indirectly measuring the intensity of chemiluminescence (CL) labels. Recently, methods using nanoparticles (NPs), in particular metal NPs, as CLIA labels have attracted considerable interest [17–19]. Among these, AuNPs have been the subject of wide research efforts directed at gene analysis [20,21] and antibody or antigen detection [22,23] because of their special properties, which include high density, large dielectric constant, and biocompatibility. It has been found that AuNPs can greatly enhance the CL intensity of the luminol-H_2_O_2_ system [24]. Accordingly, a CLIA based on irregular AuNPs was first reported by Li and co-workers [25]. However, the difficulty of controlling the synthesis of irregular nanoparticles may influence the repeatability among different batches and this limits the practical application of this method. Duan *et al*. [26] established a microplate-compatible CLIA method for the determination of human IgG based on the luminol-AgNO_3_-gold nanoparticles CL system. The CL detection is readily influenced by the sediment from the mixture of basic luminol and AgNO_3_ and has poor sensitivity. In our work, a highly sensitive immunogold amplified FI-CL immunoassay for the detection of CEA is presented through the catalytic precipitation of gold on the immunogold nanoparticles, which combined the intrinsically high sensitivity and good repeatability of FI-CL with the signal enhancement of immunogold enlargement. The present strategy shows excellent promise for ultrasensitive detection of other cancer biomarkers and infectious agents in clinical analysis.

## 2. Experimental Section

### 2.1. Reagents and Apparatus

Polystyrene 96-well microtiter plates (Costar, NY, USA) were used to perform the immunoreactions. CEA and anti-CEA antibody were purchased from R & D Systems (Minneapolis, MN, USA). Goat-anti-mouse IgG was purchased from Beijing Chengwen Company (Beijing, China). Bovine serum albumin (BSA), HAuCl_4_·3H_2_O (99.99%) was bought from Sigma-Aldrich (USA). Trisodium citrate, NaH_2_PO_4_, Na_2_HPO_4_, and NaCl are obtained from Beijing Chemical Reagents Company (Beijing, China). The luminol stock solution (2.5 × 10^−2^ M) was prepared by dissolving luminol (obtained from Sigma-Aldrich, USA) in 0.1 M NaOH solution and stored in a dark place. The buffers used were as follows: (A) coating buffer, 0.05 M carbonate/bicarbonate buffer solution, pH 9.6; (B) blocking buffer, 1% (w/v, g·mL^−1^) BSA in 0.01 M sodium phosphate buffered saline with 0.05% (v/v) Tween 20 (PBST-BSA), pH 7.4. The blocking buffer was stored at 4 °C and used within a week; (C) washing buffer, 0.01 M PBS with 0.05% (v/v) Tween 20, pH 7.4; In all the procedures, the water used was purified through an Olst ultrapure K8 apparatus (Olst, Ltd., China, resistivity >18 MΩ). All other reagents were of analytical reagent grade and used as purchased without further purification. The CL intensity was measured and recorded with an Ultra-weak luminescence analyzer and software BPCL-T15 (Institute of Biophysics, Academic Sinica, Beijing, China). Two peristaltic pumps were used to deliver all the chemicals. A six way injection valve fitted with a 100 μL sample loop was used for the injection of the sample solution. PTFE tubing (1.0 mm i.d.) was used to connect all components in the flow system. Transmission electron microscopy (TEM) was performed with a JEOL-100CX electronmicroscope (Jeol, Japan) under 80 kV accelerating voltage.

### 2.2. Preparation of Au Nanoparticles (NPs)

A solution of 15 nm AuNPs was synthesized according to a literature procedure [27] with slight modifications. Briefly, 5.0 mL of 1% trisodium citrate was quickly added to 100 mL of boiling 0.01% HAuCl_4_. Solution under reflux was stirred for 10 min, during which the color changed to red. After being slowly cooled down to room temperature, the solution was centrifuged to remove impurities and ions, and then diluted to 100 mL. The AuNPs have an average diameter of ca. 15 nm.

### 2.3. Preparation of AuNPs-Labeled Goat-Anti-Mouse IgG

The preparation of AuNPs-labeled goat-anti-mouse IgG was performed according to the modification in literature [28]. The pH of the AuNPs was adjusted to 8.0 with 0.1 M Na_2_CO_3_. Precisely, 1.0 mL of 550 μg·mL^−1^ goat-anti-mouse IgG (10% more than the minimum amount, which was determined using a flocculation test) was added to 5 mL of pH-adjusted AuNPs, followed by incubation at room temperature for 30 min. Afterward, 5% BSA was added to a final concentration of 1% with stirring about 5 min. To remove the excess of antibody, the conjugates were centrifuged at 10,000 rpm for 10 min, and the soft sediment was resuspended in 0.01 M PBS containing 1% BSA. It can be used directly or stored in 0.01 M PBS buffer with 50% glycerol for several months at −20 °C.

### 2.4. Immunoassay Procedure

The assay was performed in a polystyrene 96-well microtiter plate. Initially, 200 μL of serially diluted human CEA dilutions in coating buffer were coated on wells of the plate overnight at 4 °C. The unbound antigen was washed off three times with 300 μL of washing buffer and the uncoated active sites of polystyrene substrate were saturated with 300 μL of blocking buffer for 1 h at 37 °C, in which BSA was used as a blocking agent to prevent nonspecific adsorption of the antibody in the next step. Afterward, 200 μL of 5 μg·mL^−1^ anti-CEA antibody was added into the wells and incubated for 2 h at 37 °C. The wells were washed three times with washing buffer followed by addition of 200 μL AuNPs-labeled goat-anti-mouse IgG. Finally the wells were washed thoroughly with washing buffer (three times) and pure water (three times). Then, the wells were immediately treated with 200 μL per well of a mixture of 0.01% HAuCl_4_ and 0.4 mM NH_2_OH·HCl in a dark for 2 min at 30 °C.

### 2.5. Standard Procedures for the FI-CL Detection

**Figure 1.**
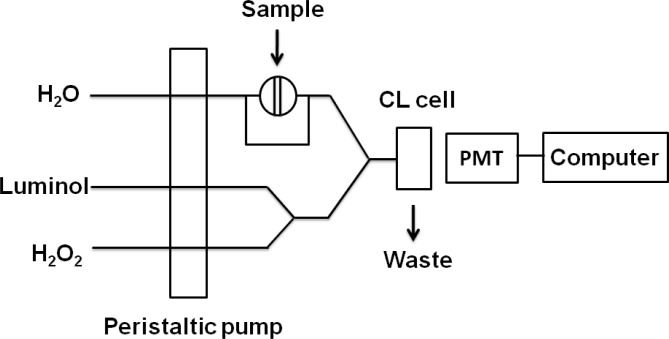
Schematic diagram of flow injection CL system.

Metallic gold on enhanced plates were washed three times with 300 μL of pure water, dried, and dissolved with 200 μL of 2.0% HNO_3_-3.4% HCl for three hours at room temperature to ensure that the Au nanoparticles were completely dissolved. Solutions were then transferred to 1.5 mL centrifuge tubes and 200 μL H_2_O and 75 μL 0.1 M sodium tartrate (C_4_H_4_O_6_Na_2_) were added to each sample in turn. Then 1.0 M NaOH was added to the AuCl_4_^−^ solutions to adjust pH. As shown in Figure 1, AuCl_4_^−^ was reacted with the mixture of luminol and H_2_O_2_ in the flow cell to produce the CL signal. The CL signals were monitored with a photomultiplier tube adjacent to the flow cell.

## 3. Results and Discussion

### 3.1. Principles of the Experiment

**Scheme 1.**
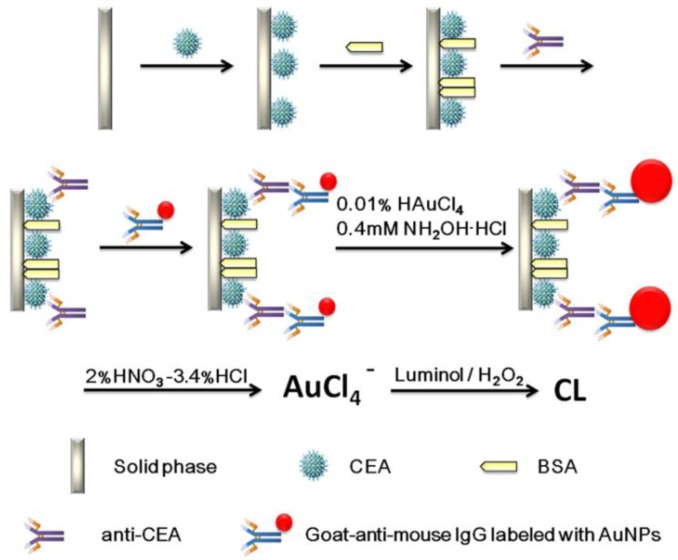
Picture representation of the proposed CL immunoassay and colloidal gold labels enlargement for human CEA.

A schematic representation of the detection principle of this noncompetitive CL immunoassay is shown in Scheme 1. The human CEA analyte is first immobilized on a 96-well polystyrene microtiter plate. The mouse-anti-CEA as primary antibody is then captured by the CEA and sandwiched by a goat-anti-mouse IgG secondary antibody labeled with colloidal gold. After that, we used a method for enlargement of colloidal Au nanoparticles called “seeding”, based on the colloidal Au surface-catalyzed reduction of Au^3+^ by NH_2_·OH. While NH_2_·OH is thermodynamically capable of reducing Au^3+^ to bulk metal, the reaction is dramatically accelerated by Au surfaces. As a result, no new particle nucleation occurs in solution, and all added Au^3+^ goes into production of larger particles. In this case, Au NPs are specifically enlarged by HAuCl_4_ and NH_2_OH·HCl for 2 min [9]. Next, the AuNPs are dissolved in a 2.0% HNO_3_-3.4% HCl solution and the gold ions (AuCl_4_^−^) were stripped out from the solid polystyrene substrate surface. The concentration of CEA was quantified based upon the concentration of dissolved AuCl_4_^−^, which was quantified by the CL intensity. A comparison of analytical performance before and after gold amplification was also investigated and the detailed optimization of the gold amplified FI-CL immunoassay is reported in the following sections.

### 3.2. Dissolution of Gold Nanoparticles

**Figure 2.**
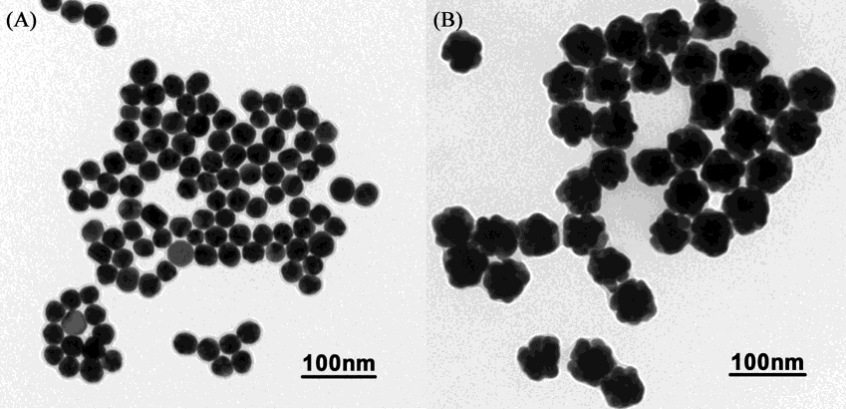
TEM images of colloidal gold before (**A**) and after (**B**) 2 min gold amplification.

**Figure 3.**
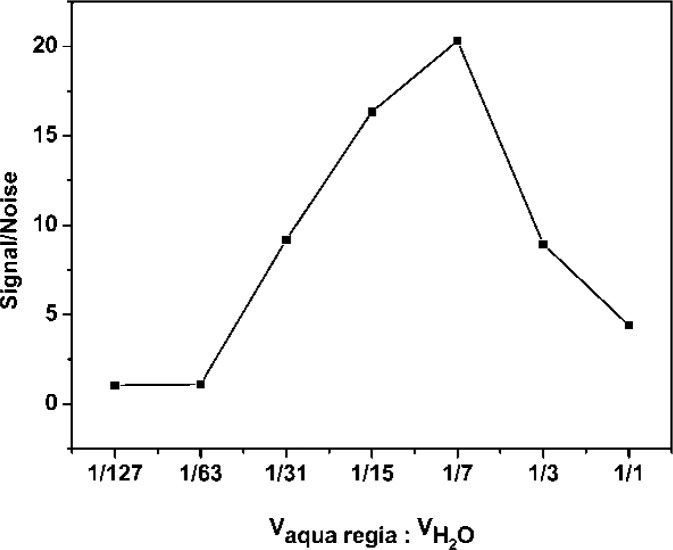
Signal/noise ratio *versus* the concentration of aqua regia. Experimental conditions: 20 μL gold nanoparticles (15 nm) was dissolved in 200 μL of different concentrations of *aqua regia*, then 200 μL H_2_O, 75 μL 0.1 M sodium tartrate and 1.0 M NaOH were added to adjust the pH of the solution to 4.

The AuNPs used in this work before and after enlargement with HAuCl_4_ and NH_2_OH·HCl are shown in Figure 2A,B. To detect the enlarged colloidal gold label by CL, the gold was dissolved to form AuCl_4_^−^, which can catalyze the luminol-H_2_O_2_ CL reaction. As the *aqua regia* (16.0% HNO_3_-27% HCl) resulted in a high CL background, dilution of the *aqua regia* was used to dissolve the colloidal gold. Therefore, the effect of the dissolution reagent concentration on CL intensity was investigated. The signal/noise ratio increased with increasing dissolution reagent concentration and reached a maximum value at 1:7 dilution of *aqua regia* (2.0% HNO_3_-3.4% HCl), as shown in Figure 3. It is possible that AuNPs could not be completely dissolved when the concentration of the dissolution reagent was lower than the 1:7 dilution of *aqua regia*. The signal/noise ratio decreased gradually when it exceeded 1:7 and it is likely that the strong ionic strength in the solution induces an increase in the CL background intensity when a much higher concentration of dissolution reagent is used. Thus, the 1:7 dilution of *aqua regia* was used as the optimum dissolution reagent for the CL assay.

**Figure 4.**
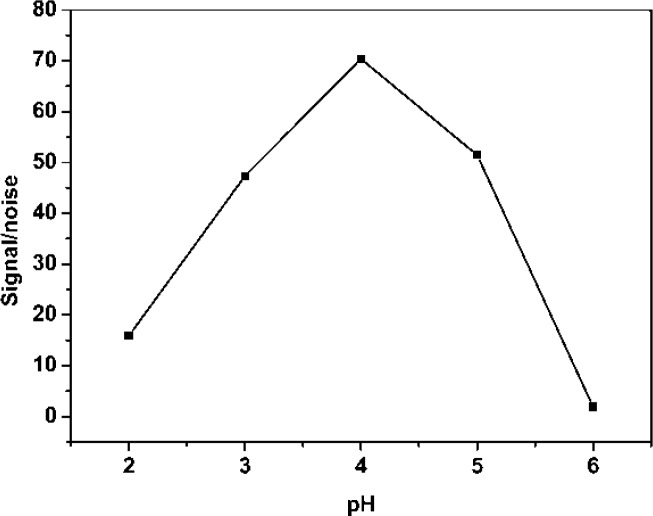
Signal/noise ratio *versus* the pH of sample. Experimental conditions: 20 μL gold nanoparticles (15 nm) was dissolved in 200 μL of *aqua regia* (2.0% HNO_3_-3.4% HCl), then 200 μL H_2_O, 75 μL 0.1 M sodium tartrate and 1.0 M NaOH were added to adjust the pH of the solution.

The catalytic activity of AuCl_4_^−^ on the luminol-H_2_O_2_ CL reaction is greatly influenced by the pH of the AuCl_4_^−^ solution. Therefore, the effect of AuCl_4_^−^ pH on CL intensity was investigated by using 1.0 M NaOH and 0.1 M C_4_H_4_O_6_Na_2_ to adjust the acidity of the solution after dissolving the AuNPs with 1:7 (v/v) *aqua regia*. As shown in Figure 4, the signal/noise ratio increased with increasing pH of sample solution because the blank CL intensity decreased with increasing sample pH below 4.0, reaching the highest value at pH 4.0. However, when the pH of the sample was higher than 4.0, the signal/noise ratio decreased. The cause is that the stability of AuCl_4_^−^ has been destroyed. Therefore, pH 4.0 was chosen as the optimum pH value for the CL measurement.

### 3.3. Optimization of the CL Detection Conditions

To optimize the proposed CL assay, the effects of luminol and H_2_O_2_ pH on the CL intensities were studied. The optimization of the pH value of luminol was investigated over the pH range 10.0–13.0. The result indicates that the signal/noise ratio had a maximum at pH 12.5. The effects of luminol and H_2_O_2_ concentration on the CL intensity of the sample and blank were also examined. This indicated that the CL intensity increased as the luminol and H_2_O_2_ concentrations were increased but the CL intensity of the blank became very high when the concentration of luminol exceeded 1.0 × 10^−4^ M and led to poor reproducibility. Considering the CL intensity and the consumption of the reagents, 7.5 × 10^−5^ M and 0.01 M H_2_O_2_ were chosen for subsequent work (data not shown).

### 3.4. Analytical Performance

**Figure 5.**
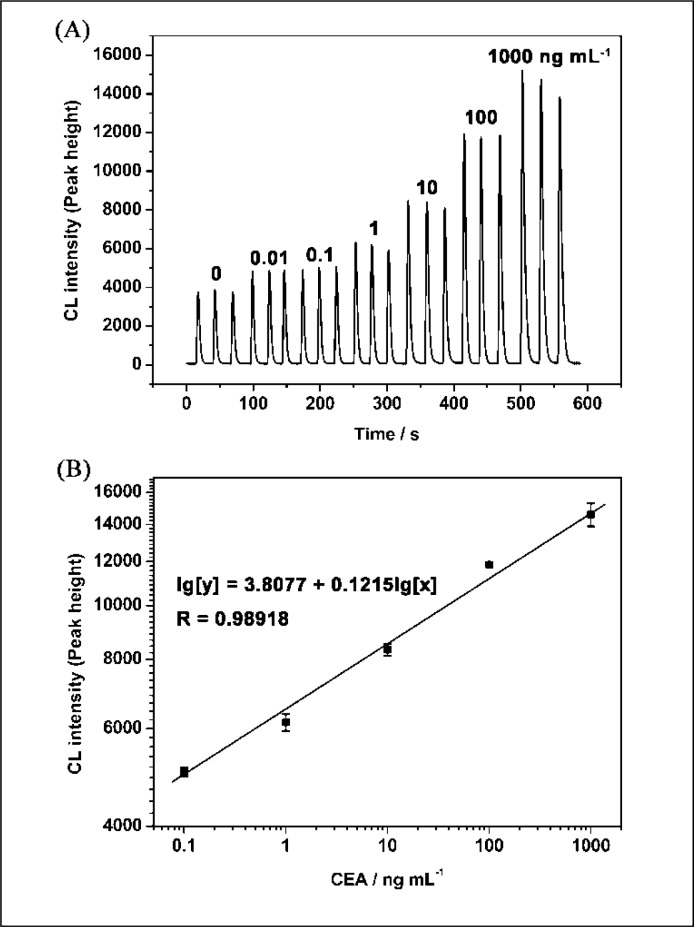
Relationship between the concentration of CEA and CL intensity after the immunogold enlargement.

Under the optimum conditions described above, the relationship between CEA concentration and CL intensity was investigated. The results showed that the CL intensities of luminol-H_2_O_2_-AuCl_4_^−^ increased with the increase of concentration of the CEA ranging from 10 pg·mL^−1^ to 1 μg·mL^−1^ (Figure 5A). The linear range for CEA was 100 pg·mL^−1^ to 1 μg·mL^−1^ with the equation of lg[y] = 3.8077 + 0.1215l g[x] (y is the CL intensity; x is the concentration of CEA, ng·mL^−1^; n = 3, R = 0.9892; Figure 5B). The relative standard deviations for 10 pg·mL^−1^ and 1 μg·mL^−1^ of CEA were 0.44% and 4.7%, respectively (n = 3). The detection limit was 20 pg·mL^−1^. Table 1 shows the comparison between the proposed CL method and general immunoassay formats for determination of CEA. It can be seen that the proposed CLIA is competitive with or better than other immunoassay formats and has the advantage of simple instrumentation.

## 4. Conclusions

The feasibility of a highly sensitive FI-CL immunoassay, based on the quantitative enlargement of immunogold tags, has been demonstrated. AuNPs are dissolved into Au^3+^, which catalyzes the luminol chemiluminescence (CL) reaction. The CL intensity, which is proportional to the amount of CEA, could be greatly enhanced. The response of this immunosensor was linear from 0.1–1,000 ng·mL^−1^ with a LOD of 20 pg·mL^−1^ (S/N = 3). The procedure involved in this work is simple, low cost and rapid. The proposed method can provide high sensitivity, a wide linear range and represents a new approach to the ultrasensitive determination of other bioactive molecules for early disease diagnosis.

## Figures and Tables

**Table 1.** Comparison of various immunoassay methods for CEA determination.

**Label**	**Analytical method**	**Detection limit (ng·mL^−1^)**	**Reference**
HRP	ELISA	0.048	[29]
HRP	Chemiluminescence immunoassay	0.5	[30]
HRP	Enzyme-linked immunoassay	3.0	[31]
AuNPs	ICPMS immunoassay after silver amplification	0.03	[32]
AuNPs	Electrochemical immunoassay	0.27	[33]
AuNPs	Electrochemical immunoassay	0.57	[34]
AuNPs	Chemiluminescence immunoassay after gold enlargement	0.02	this work
AuNPs	Chemiluminescence immunoassay	1.0	this work
